# Genetic rescue of an endangered domestic animal through outcrossing with closely related breeds: A case study of the Norwegian Lundehund

**DOI:** 10.1371/journal.pone.0177429

**Published:** 2017-06-01

**Authors:** Astrid V. Stronen, Elina Salmela, Birna K. Baldursdóttir, Peer Berg, Ingvild S. Espelien, Kirsi Järvi, Henrik Jensen, Torsten N. Kristensen, Claudia Melis, Tommaso Manenti, Hannes Lohi, Cino Pertoldi

**Affiliations:** 1 Section of Biology and Environmental Science, Department of Chemistry and Bioscience, Aalborg University, Aalborg Øst, Denmark; 2 Research Programs Unit, Molecular Neurology and Department of Veterinary Biosciences, University of Helsinki, and Folkhälsan Research Center, Helsinki, Finland; 3 Department of Biosciences, University of Helsinki, Helsinki, Finland; 4 Icelandic Genetic Resource Center, Hvanneyri, Borganes, Iceland; 5 Nordic Genetic Resource Center, Ås, Norway; 6 Norwegian Lundehund Club, Tangen, Trondheim, Norway; 7 Centre for Biodiversity Dynamics, Department of Biology, Norwegian University of Science and Technology, Trondheim, Norway; 8 Department of Biosciences, Section for Genetics, Ecology and Evolution, Aarhus University, Aarhus C, Denmark; 9 Queen Maud University College of Early Childhood Education, Trondheim, Norway; 10 Aalborg Zoo, Aalborg, Denmark; University of Florence, ITALY

## Abstract

Genetic rescue, outcrossing with individuals from a related population, is used to augment genetic diversity in populations threatened by severe inbreeding and extinction. The endangered Norwegian Lundehund dog underwent at least two severe bottlenecks in the 1940s and 1960s that each left only five inbred dogs, and the approximately 1500 dogs remaining world-wide today appear to descend from only two individuals. The Lundehund has a high prevalence of a gastrointestinal disease, to which all remaining dogs may be predisposed. Outcrossing is currently performed with three Nordic Spitz breeds: Norwegian Buhund, Icelandic Sheepdog, and Norrbottenspets. Examination of single nucleotide polymorphism (SNP) genotypes based on 165K loci in 48 dogs from the four breeds revealed substantially lower genetic diversity for the Lundehund (H_E_ 0.035) than for other breeds (H_E_ 0.209–0.284). Analyses of genetic structure with > 15K linkage disequilibrium-pruned SNPs showed four distinct genetic clusters. Pairwise F_ST_ values between Lundehund and the candidate breeds were highest for Icelandic Sheepdog, followed by Buhund and Norrbottenspets. We assessed the presence of outlier loci among candidate breeds and examined flanking genome regions (1 megabase) for genes under possible selection to identify potential adaptive differences among breeds; outliers were observed in flanking regions of genes associated with key functions including the immune system, metabolism, cognition and physical development. We suggest crossbreeding with multiple breeds as the best strategy to increase genetic diversity for the Lundehund and to reduce the incidence of health problems. For this project, the three candidate breeds were first selected based on phenotypes and then subjected to genetic investigation. Because phenotypes are often paramount for domestic breed owners, such a strategy could provide a helpful approach for genetic rescue and restoration of other domestic populations at risk, by ensuring the involvement of owners, breeders and managers at the start of the project.

## Introduction

Genetic rescue–augmentation of genetic variation in depauperate populations–has occurred by means of natural experiments or initiated by humans, and appears to have resulted in favourable outcomes at least in the short term (e.g. [1–4]). By population we here refer to a group of individuals interbreeding naturally or by human management, including domestic animal breeds. For populations at risk, outcrossing with individuals from a related but different population may sometimes be the only option left to avoid extinction. These efforts can involve costs and benefits in terms of changes in phenotype such as morphology, behaviour, and local adaptations, and a general increase in genetic variation and decrease in inbreeding [5–9]. Such changes, should they occur, might nonetheless be advantageous compared to the alternative of no action and possible extinction. Accordingly, genetic rescue may be considered the best solution for breeds suffering due to low levels of genetic variation and high levels of inbreeding.

The endangered Norwegian Lundehund (henceforth Lundehund) is undergoing genetic rescue and provides a constructive example as a model system, because dog genomic resources are readily available and comparatively inexpensive, and the demographic history of dogs is relatively well-known. The Lundehund breed is a small Spitz from coastal Norway where it was traditionally used to hunt Atlantic puffins (*Fratercula arctica*, hereafter puffins). The Lundehund population underwent at least two severe bottlenecks in the 1940s and 1960s where each event appears to have left only five inbred individuals [10–12]. The around 1500 dogs remaining at present appear to descend from only two individuals [13]. Accordingly, the Lundehund is now highly inbred [11–14]: for example [14] reported an inbreeding coefficient (F_IS_) equal to 0.87 based on 26 microsatellite loci genotyped in 125 Lundehund individuals. The Lundehund is no longer used to hunt puffins, a species now considered vulnerable on the Norwegian mainland (http://www.artsportalen.artsdatabanken.no/#/Fratercula+arctica/3611). However, the Lundehund exhibits a number of unique morphological features associated with its historical function. These include enhanced neck and shoulder joint flexibility, polydactyly (additional digits) and “sealable” ears, which together with small body size appear to have permitted easier access to, and mobility in, the steep coastal cliffs where the puffins nest [10, 12].

Lundehund individuals are reported to have a high prevalence of gastroenteropathy, a disease of the stomach and intestines [15–16], which influences individual health and survival [10], and is financially costly to dog owners. The prevalence of gastroenteropathy is unknown but assumed to be high. Its clinical signs include intermittent diarrhea, vomiting, weight loss, lethargy, ascites (accumulation of fluid in the peritoneal cavity causing abdominal swelling) and subcutaneous edema (swelling caused by excess fluid trapped in body tissues) mostly of the hind legs ([15] and references therein). Unpublished data from the Norwegian Lundehund Club (http://web2.nkk.no/filestore/RAS/RAS-Norsk-lundehund-ver-1.pdf, p.17) suggest that approximately 30% of Lundehund individuals die from a type of gastroenteropathy known as intestinal lymphangiectasia, commonly known as IL. The high prevalence of gastrointestinal disease is believed to be an example of inbreeding depression [11]. Although the genetic basis underlying gastroenteropathy is unknown, an increase in genetic variation by outcrossing with unaffected individuals from related breeds is likely to improve Lundehund health, which will advance dog welfare. Consequently, careful selection of healthy individuals produced from outcrossing could offer immediate benefits. A broader gene pool may also help researchers to identify the genetic mechanism(s) underlying the gastroenteropathy, which is currently difficult as the allele(s) at the gene(s) causing the disease is believed to be fixed in the population.

Polydactyly, a signature Lundehund trait, has been investigated across taxa for possible negative effects on health and development in vertebrates by means of pleiotropic effects whereby one gene influences two or more apparently unrelated phenotypic traits [17]. However, the extent to which these problems are directly linked to polydactyly, or independent consequences of inbreeding, is unresolved [17]. In an analysis of runs of homozygosity (ROH) in the Lundehund, [12] reported two genes, BMPR1B on chromosome 32 and PRRX2 on chromosome 9, previously reported as associated with polydactyly in mice. Furthermore, an intronic mutation in the preZRS of the LMBR1 gene on chromosome 16 has been implicated in polydactyly in several dog breeds including Lundehund [13, 18]. The Lundehund breed with low genetic variability may be a model population for the study of polydactyly [13], and further research could help illuminate the genetic basis for this feature. Following outcrossing with other breeds, some offspring will likely not carry the trait. Carriers and non-carriers may then be compared over time to examine whether this trait, previously valued for hunting, now might represent a cost, e.g., in the form of reduced survival, for the breed and its long-term conservation.

The selection of breeds and individuals for outcrossing, and the procedures for managing the breeding plans, is a balance between infusing new genetic variation relatively quickly, and careful monitoring to ensure that what remains of the original genetic variability is not swamped. Moreover, the breeding program aims to ensure that morphological, behavioural and other breed characteristics are conserved as much as possible. Outcrossing is currently ongoing and breeding has been undertaken/planned with all three candidate breeds. The objective of this study is to investigate 1) the population genetic structure and differentiation among the Lundehund and candidate breeds for outcrossing, and 2) the occurrence of outlier loci among breeds, the presence of which may indicate selection on genes in flanking regions and thus adaptive differences among breeds that, over time, could benefit Lundehund recovery and preservation. Humans have likely performed selective breeding in the Lundehund and candidate breeds according to their historical role in e.g. herding and hunting, including the unique adaptations to puffin hunting found in the Lundehund. Selective breeding by humans may be expected to produce outlier loci among breeds. However, as all four breeds originate from relatively similar Nordic environments we do not expect such differences to represent naturally selected traits that carry a risk of being maladaptive in crossbred individuals. The main aim of the outlier analyses is to help clarify whether there may be signs of standing genetic variation from adaptation or artificial selection among the candidate breeds, indicating that each breed could contribute unique and potentially adaptive genetic variation to the depauperate Lundehund genome. Importantly, the bottlenecks experienced by the Lundehund may have augmented the probability of finding outliers between this breed and the candidate breeds owing to genetic drift. However, if outlier SNPs near genes believed to be associated with health and survival occur among all four breeds, or pairwise between candidate breeds, this would appear to support the inclusion of multiple candidate breeds in the programme to augment Lundehund genetic variation and evolutionary potential.

## Materials and methods

We genotyped 48 dogs including 17 Lundehund and individuals from three candidate breeds for crossbreeding: 10 Norwegian Buhund (henceforth Buhund), nine Icelandic Sheepdogs, and 12 Norrbottenspets, a breed originating from northern Fennoscandia and Russian Karelia and named after the Norrbotten province in northern Sweden. The three candidate breeds to be used for crossbreeding have been chosen by the Norwegian Lundehund Club (http://lundehund.no/index.php/krysningsprosjektet) based on morphology, behaviour, shared history and (assumed) high genetic relatedness to the Lundehund breed. However, the individuals in this study were not expected to contribute directly to the breeding program.

Collection of Norrbottenspets and Buhund samples was approved by the Animal Ethics Committee at the State Provincial Office of Southern Finland (ESLH-2009-07827/Ym-23 and ESAVI/6054/04.10.03/2012). Icelandic Sheepdog blood samples were collected by a veterinarian, and Lundehund individuals were sampled by non-invasive buccal swabs. Blood or buccal swab samples were genotyped with the Canine HD Bead Chip (Illumina) with 172 115 SNPs, and we performed quality screening in GenomeStudio (Illumina) following the program guidelines (http://www.illumina.com/Documents/products/technotes/technote_infinium_genotyping_data_analysis.pdf). Individual call rates were ≥ 0.98, except for one Icelandic Sheepdog with call rate 0.91. Subsequently, we imported the data into PLINK [19] (v1.07 and 1.9) and calculated genetic diversity parameters; expected (H_E_) and observed heterozygosity (H_O_), missingness (missing genotype rate), polymorphism (P) and identity-by-descent (IBD) within and among breeds. Next we screened the data across all breeds with criteria of minor allelic frequency 0.01 (the minor allele must be present in at least 1% of the sample; PLINK command—maf 0.01) and genotyping success rate 0.98 (genotypes at a given SNP must be present in 98% of the samples or more; PLINK command—geno 0.02). We then removed sex-linked SNPs (screening only the autosomal chromosomes) and pruned the data for markers in linkage disequilibrium (LD) with PLINK command—indep 50 5 2, where 50 is the size of the sliding window, 5 is the number of SNPs shifted in each step, and 2 is the variance inflation factor (VIF), which is a measure of multicollinearity where two or more predictor variables in a multiple regression are highly correlated. The VIF is 1/(1-R^2^) where R^2^ represents the multiple correlation coefficient for a SNP simultaneously regressed on all other SNPs. The PLINK guidelines recommend using a VIF from 1.5 to 2.0 for small sample sizes to avoid removing too many SNPs.

We investigated genetic clustering of the data with ADMIXTURE [20], which employs a cross-validation procedure to help identify the optimal value for the number of population clusters (K). A supported K-value is one which has low cross-validation error relative to alternate K-values [21]. We investigated a range of population clusters (K) from 1–6, with 20 cross-validations for each K-value and 1000 bootstrap replicates. Thereafter, we performed analyses with equalized sample sizes to examine whether the larger number of Lundehund dogs assessed compared to sample sizes in the other breeds may influence the results. We then performed a principal component analysis (PCA) with the same LD-pruned data set to examine the distribution of breeds and individuals with the *adegenet* package [22] in R 2.14.2 [23].

We calculated pairwise F_ST_ [24] among breeds for the Lundehund and the three candidate breeds in PLINK and obtained p-values for the null hypothesis of no differentiation for the estimates by permutations. To provide additional information for the genetic rescue project, we also calculated pairwise F_ST_ between Lundehund and available samples of 10 additional Nordic Spitz breeds (E. Salmela and H. Lohi, unpublished data).

Subsequently, we examined the data with BayeScan [25] to identify outlier loci–where differences in allele frequencies between breeds were more divergent than those expected under the neutral distribution–that may indicate genes or areas of the genome under potential selection. We ran one analysis across all four breeds, and subsequently performed six pairwise comparisons between the breeds. In BayeScan, the choice of prior value for the neutral model represents a trade-off between finding loci under (weak) selection and failing to recognize genuine outliers from loci at the extreme end of the natural distribution [26]. However, preliminary tests with priors of 10 and 100 identified the same loci as outliers (though the order of loci differed somewhat) and we retained the value of 10. The decay of LD in the Lundehund is very slow with r^2^ = 0.95 in SNPs 50 kb apart [12]. For outlier loci, we therefore examined wide flanking regions of the canid genome, 1 megabase (Mb) or 1 million bases on both sides, in the NCBI Genome Browser (http://www.ncbi.nlm.nih.gov/genome?term=canis%20lupus%20familiaris) for genes under possible selection. Subsequently, we examined the data in LOSITAN [27–28] to evaluate if loci identified by BayeScan would again be highlighted as outliers under either divergent or balancing selection. We did a first test with all individuals, and then a second test with five randomly selected individuals per breed to examine the effect of a simulated bottleneck. Because it was not possible to run the entire data set at once, we did four runs with 4K loci each and 10K simulations per run. The restricted sample size is expected to provide a more conservative test as fewer loci are available for defining the neutral distribution, i.e., loci not under selection. For populations such as the Lundehund that have been severely affected by genetic drift it may be particularly challenging to separate effects of genetic drift and selection, and we examined the data for ROH to compare these results with findings from outlier tests. ROH were analyzed in PLINK using the functions—homozyg and—homozyg-group with their default parameters. This analysis aims to locate relatively long stretches of consecutive homozygous markers in each individual (allowing, with the default values, for one heterozygous and five missing genotype calls within windows of 5 Mb and 50 SNPs), and summarizes such regions shared between the individuals. The analysis was done on the dataset unpruned for LD but including monomorphic markers; X-chromosomal SNPs were excluded.

Finally, we explored genetic diversity along the chromosomes to detect potential islands of divergence. In this assessment we used all chromosomes including the X-chromosome. We calculated the Pearson correlation coefficient for allele frequencies between the Lundehund and each of the three candidate breeds, and plotted the mean the coefficient *versus* SNP position in sliding windows for every 20 and 100 SNPs within each chromosome with 95% confidence intervals. Next, we excluded the Lundehund and plotted the Norrbottenspets, believed to be the most genetically diverse, *versus* the two other candidate breeds. We subsequently examined expected and observed heterozygosity for each of the four breeds.

## Results

We obtained profiles containing 165 293 (165K) SNPs and, after application of filters for genotyping success and minor allele frequency, 119 920 high-quality SNPs remained. Subsequent pruning for loci in linkage disequilibrium and exclusion of sex-linked SNPs resulted in a data set of 15 648 autosomal unlinked loci. Genetic diversity was substantially lower for the Lundehund than for the other breeds (Table 1). The Norrbottenspets exhibited the highest genetic diversity; the Buhund and the Icelandic Sheepdog had intermediate and comparable values. Polymorphism values revealed a similar pattern, whereas the order was opposite for IBD with very high values for the Lundehund, lower and relatively similar values for the Buhund and the Icelandic Sheepdog, and Norrbottenspets at the lower end. In calculations of IBD across the entire sample with 165K SNPs, all comparisons of individuals from different breeds gave IBD values of zero.

**Table 1:** Genetic diversity measures for the endangered Norwegian Lundehund and three candidate breeds for genetic rescue.

Breed	H_O_ (S.E.)^1^	H_E_ (S.E.)^2^	Percent polymorphic loci	Percent missing loci	IBD^3^ Mean (range)	ROH^4^ Mean (range)
Lundehun (n = 17)	0.038 (0.0003)	0.035 (0.0003)	10.52	0.73	0.899 (0.842–0.954)	2053.1 (2003.3–2094.2)
Buhund (n = 10)	0.230 (0.0006)	0.217 (0.0005)	64.06	0.41	0.365 (0.307–0.465)	674.0 (449.7–857.2)
Icelandic Sheepdog (n = 9)	0.232 (0.0006)	0.209 (0.0005)	62.19	1.40	0.389 (0.276–0.525)	657.5 (340.6–837.4)
Norrbottenspets (n = 12)	0.298 (0.0005)	0.284 (0.0005)	80.18	0.58	0.210 (0.152–0.310)	190.1 (59.3–312.6)

^1^Observed heterozygosity (H_O_) with standard error (S.E.)

^2^Expected heterozygosity (H_E_) with standard error (S.E.)

^3^Mean values for identity-by-descent (IBD) between pairs of individuals calculated in PLINK.

^4^Runs of homozygosity (ROH) in Mb per individual, calculated in PLINK.

**Fig 1:**
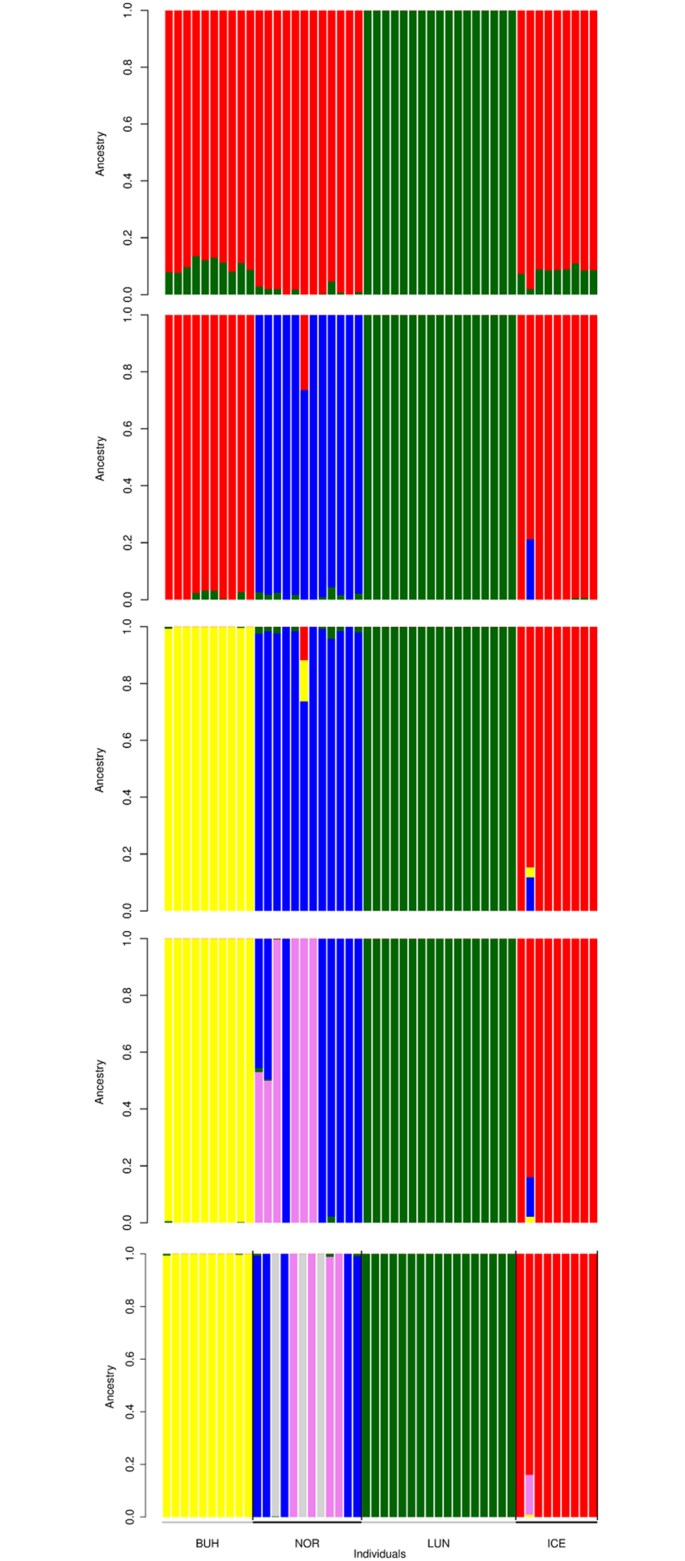
ADMIXTURE results for n = 48 Nordic dogs with K = 2–6 population clusters. Analyses with 15 648 single nucleotide polymorphism (SNP) loci for the four breeds Norwegian Lundehund (LUN), Norwegian Buhund (BUH), Icelandic Sheepdog (ICE) and Norrbottenspets (NOR). Comparison of cross-validation errors (Figure A in S1 File) showed highest support for K = 2, with K = 4 having nearly the same support.

**Fig 2:**
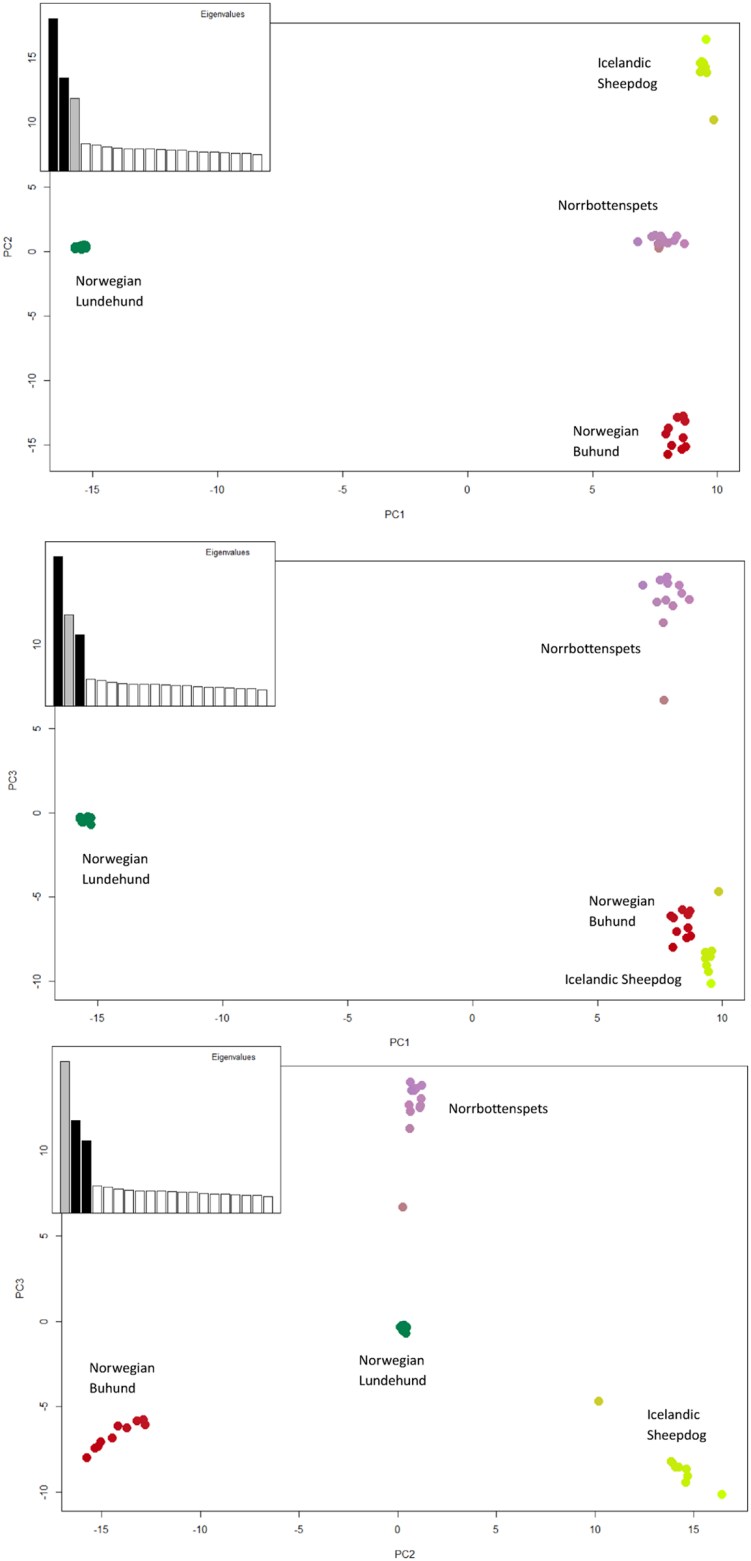
Principal component analyses for n = 48 Nordic dogs with 15 648 single nucleotide polymorphism markers. Norwegian Buhund (n = 10), Norrbottenspets (n = 12), Norwegian Lundehund (n = 17), and Icelandic Sheepdog (n = 9). The plots show the first three principal component (PC) axes, where PC axis 1, 2 and 3 represents respectively 17.4, 10.6 and 8.3% of the variation.

The cross-validation errors for the ADMIXTURE results were lowest for K = 2 (0.34, Figure A in S1 File) separating the Lundehund individuals from all other breeds, followed by K = 4 (0.35) that identified each breed separately (Fig 1). Higher values of K displayed within-breed diversity in the Norrbottenspets. Analyses with equalized sample sizes gave the same results (Figure B in S1 File). The PCA results were consistent with ADMIXTURE in showing all four breeds as separate clusters (Fig 2), where the Lundehund appeared as the most isolated and a highly uniform group on the axis of PC 1 that represented 17.4% of the variation.

Pairwise F_ST_ values between Lundehund and the three candidate breeds were highest for Icelandic Sheepdog, followed by Buhund and Norrbottenspets, and the Norrbottenspets exhibited the lowest values across comparisons (Table 2). Based on F_ST_ values calculated among the Lundehund and the three candidate breeds, the Lundehund is closer to the Norrbottenspets than to the Buhund and least related to the Icelandic Sheepdog. Comparisons between the Lundehund and additional Nordic Spitz breeds (Table A in S1 File) showed unweighted F_ST_-values between the Lundehund and the three candidate breeds ranging from 0.166 to 0.247. However, the results also suggested that certain breeds not chosen as crossbreeding candidates (e.g. Finnish Lapphund, Karelian Beardog) appeared to be genetically closer, with F_ST_-values of 0.128 and 0.211, respectively, although small sample sizes prevent a full comparison for some breeds. In contrast, another breed from Norway, the Norwegian Elkhound (here represented by the grey variety) is genetically more distant from Lundehund than several breeds originating from outside Norway.

**Table 2:** Pairwise F_ST_-values for Lundehund and three candidate breeds for genetic rescue.

Dog breed	Buhund (n = 10)	Icelandic Sheepdog (n = 9)	Norrbottenspets (n = 12)
Lundehund (n = 17)	u: 0.228 (0.0027) w: 0.381 (0.0055) perm:363	u: 0.247 (0.0055) w: 0.410 (0.0082) perm: 364	u: 0.166 (0.0054) w: 0.263 (0.0054) perm:367
Buhund (n = 10)	—	u: 0.120 (0.0087) w: 0.200 (0.0087) perm:229	u: 0.075 (0.0081) w: 0.120 (0.0027) perm:369
Icelandic Sheepdog (n = 9)	—	—	u: 0.074 (0.0146) w: 0.122 (0.0097) perm:205

Values are presented as unweighted (u) and weighted (w) with p-value in parentheses and the number of permutations (perm).

BayeScan results across all four breeds and from pairwise comparisons highlighted 18 outlier SNPs (Table 3; Table B in S1 File), ten of which were observed in comparisons across all four breeds. Examination of flanking regions showed genes known or believed to be associated with immune function, metabolism, physical development, cognition and sensory function (Table 3). The gene *LRRTM1* (chromosome 17), which was observed in the flanking region for an outlier SNP in the pairwise test for Buhund–Norrbottenspets, has previously been related to a rare phenotype in mice associated with avoiding confined space [29]. *POC1A* (chromosome 20) has been connected to bone formation, short stature and facial dysmorphism in humans [30], and was detected in the outlier test across all four breeds. *GLB1* (chromosome 23) was observed in the flanking region of an outlier in the test across all four breeds as well as in two pairwise tests (Buhund–Norrbottenspets; Icelandic Sheepdog–Norrbottenspets); mutations in this gene have been linked to metabolic diseases and hypermobile joints [31]. No outliers were detected in pairwise comparisons involving the Lundehund and other breeds.

**Table 3:** Outlier single nuclear polymorphism (SNP) loci detected in BayeScan and summary^1^ of key functional genes.

Chr and SNP position^2^ (bp)	SNP ID	BayeScan log_10_(PO)^3^	BayeScan FDR^4^	Gene(s)	Function summary (distance^5^ to outlier SNP)
					**IMMUNE FUNCTION / DISEASE**
Chr3:54160905	BICF2S23152168	0.553 (4B)	0.113 (4B)	*MFGE8*	Wound healing, autoimmune disease, and cancer (0.78 Mb).
Chr4:61093673	BICF2P480043	1.119 (4B) 0.894 (BN)	0.039 (4B) 0.076 (BN)	*GM2A*	Tay-Sachs disease (0.12 Mb).
Chr4:61093673	BICF2P480043	1.119 (4B) 0.894 (BN)	0.039 (4B) 0.076 (BN)	*TNIP1*	Autoimmunity, tissue homeostasis. Mutations associated with arthritis and systemic lupus erythematosus (0.29 Mb).
Chr4:61093673	BICF2P480043	1.119 (4B) 0.894 (BN)	0.039 (4B) 0.076 (BN)	*CD74*	Class II major histocompatibility complex (MHC) (0.80 Mb).
Chr10:70696871	BICF2P663203	0.916 (4B)	0.061 (4B)	*PROKR1*	Inflammation, possible role in Hirschsprung's disease, a condition that affects the large intestine (colon) (0.01 Mb).
Chr10:70696871	BICF2P663203	0.916 (4B)	0.061 (4B)	*GKN1*	Gastric cancer (gastric mucosa) (0.25 Mb).
Chr10:70696871	BICF2P663203	0.916 (4B)	0.061 (4B)	*ANTXR1*	Colorectal cancer (0.38 Mb).
Chr14:6283930	BICF2G630518318	2.152 (IN)	0.007 (IN)	*AKR1B1*	Diabetes (0.33 Mb).
Chr23:6159976	TIGRP2P309625_rs8576070	3.699 (4B) 0.763 (BN) 0.593 (IN)	0.000 (4B) 0.086 (BN) 0.108 (IN)	*CCR4*	Immune function, canine atopic dermatitis (AD) (0.70 Mb).
					**METABOLISM/DIGESTION**
Chr4:61093673	BICF2P480043	1.119 (4B) 0.894 (BN)	0.039 (4B) 0.076(BN)	*NMUR2*	Gut and central nervous system; regulation of food intake and body weight (0.89 Mb).
Chr23:6159976	TIGRP2P309625_rs8576070	3.699 (4B) 0.763 (BN) 0.593 (IN)	0.000 (4B) 0.086 (BN) 0.108 (IN)	*LOC485570/ABHD5*	Chanarin-Dorfman syndrome (triglyceride storage disease with impaired long-chain fatty acid oxidation) (0.55 Mb).
					**PHYSICAL DEVELOPMENT**
Chr3_54160905	BICF2S23152168	0.553 (4B)	0.113 (4B)	*ACAN*	Cartilagenous tissue and compression in cartilage (0.71 Mb).
Chr4:61093673	BICF2P480043	1.119 (4B) 0.894 (BN)	0.039 (4B) 0.076 (BN)	*MYOZ3*	Skeletal muscle (0.60 Mb)
Chr4:61093673	BICF2P480043	1.119 (4B) 0.894 (BN)	0.039 (4B) 0.076 (BN)	*TCOF1*	Treacher-Collins syndrome in humans; the dog homolog is associated with brachycephaly (broad skull/short face) (0.83 Mb)
Chr13:22691061	BICF2P1052982	0.758 (BN)	0.102 (BN)	*HAS2*	Wrinkled and thickened skin (Shar-pei dogs); strong selection for the skin phenotype seems to enrich for a pleiotropic mutation predisposing dogs to a periodic fever syndrome (0.67 Mb).
Chr17:46888130	TIGRP2P232559_rs8827295	0.681 (BN)	0.127 (BN)	*LOC100687463/REG3G*	Skeletal muscle, peripheral nerve regeneration (1.01 Mb).
Chr20:40918366	BICF2P941107	1.198 (4B)	0.028 (4B)	*POC1A*	Bone, hair, and nail formation; mutations associated with short stature (0.24 Mb).
Chr23:6159976	TIGRP2P309625_rs8576070	3.699 (4B) 0.763 (BN) 0.593 (IN)	0.000 (4B) 0.086 (BN) 0.108 (IN)	*CRTAP*	Skeletal development; defects associated with osteogenesis imperfecta, a connective tissue disorder characterized by bone fragility and low bone mass (0.56 Mb).
Chr23:6159976	TIGRP2P309625_rs8576070	3.699 (4B) 0.763 (BN) 0.593 (IN)	0.000 (4B) 0.086 (BN) 0.108 (IN)	*GLB1*	GM1-gangliosidosis (progressive generalized neurodegeneration and mild skeletal changes), and Morquio B syndrome (metabolic disease where the body is unable to break down long chains of sugar molecules called glycosaminoglycans; symptoms include hypermobile joints) (0.58 Mb).
					**BRAIN/COGNITION**
Chr4:61093673	BICF2P480043	1.119 (4B) 0.894 (BN)	0.039 (4B) 0.076 (BN)	*CAMK2A*	Spatial and contextual learning, circadian behavioural activities (0.94 Mb).
Chr5:22167407	BICF2P261357	0.566 (4B)	0.082 (4B)	*HTR3A*, *HTR3B*	Receptor for serotonin (biogenic hormone that functions as a neurotransmitter) (0.17 Mb, 0.22 Mb).
Chr5:22167407	BICF2P261357	0.566 (4B)	0.082 (4B)	*DRD2*	Neurotransmitter, helps control the brain's reward and pleasure centers, movement, emotion (0.63 Mb).
Chr17:46888130	TIGRP2P232559_rs8827295	0.681 (BN)	0.127 (BN)	*LRRTM1*	LRRTM1-deficient mice: rare phenotype of avoiding small enclosures (claustrophobia-like behaviour); humans: Schizophrenia (0.01 Mb).
Chr17:46888130	TIGRP2P232559_rs8827295	0.681 (BN)	0.127 (BN)	*CTNNA2*	Excitement-seeking (0.17 Mb)
					**SENSORY FUNCTION**
Chr14:6283930	BICF2G630518318	2.152 (IN)	0.007 (IN)	*OR2M9*^6^, *OR2T1*, *OR2T2*	olfactory receptors (0.98 Mb, 0.72 Mb, 0.64 Mb). (Also nearby are LOC482235, LOC482236, LOC10068735, LOC6078, LOC482239, COR2T18, LOC100686566, LOC482243, LOC100686790, LOC607953, cOR2G5)

^1^Outlier single nuclear polymorphism (SNP) loci detected in BayeScan across four dog breeds and summary of key functional genes found in the flanking regions (1 megabase in either direction) of the canine genome. Function summary is based on references from the NCBI database (http://www.ncbi.nlm.nih.gov/gene). Analyses are based on the dog breeds Norwegian Lundehund (n = 17), Norwegian Buhund (n = 10), Icelandic Sheepdog (n = 9) and Norrbottenspets (n = 12) and 15 648 autosomal unlinked SNP loci.

^2^SNP positions in the Canine Illumina HD Bead Chip and shown here are provided according to genome annotation CanFam2, and were lifted (http://genome.ucsc.edu/cgi-bin/hgLiftOver) to the most recent genome annotation CanFam3 for assessment.

^3^Pairwise breed comparisons for: B–Buhund; N–Norrbottenspets; I–Icelandic Sheepdog. 4B: across all four dog breeds, including Lundehund.

^4^False discovery rate threshold (q-value).

^5^Approximate distance from center of gene to outlier SNP.

^6^Labeled OR2M5 in CanFam2.

Although the genetic findings from the abovementioned studies have no known direct link to the Lundehund or the candidate breeds, the among-breed variation observed in the outlier tests appears relevant to the conservation breeding program, considering Lundehund history, human-directed selection, current breed standard, and health concerns. The LOSITAN results for all individuals were consistent with BayeScan in identifying the 18 SNPs as outliers under divergent selection, with the probability of the sample F_ST_ exceeding simulated F_ST_ being above 0.99. For the test of five dogs per breed, 16 SNPs were clear outliers (probability > 0.96) whereas two SNPs, BICF2P663203 (chromosome 10) and BICF2P993491 (chromosome 17), had probability values of 0.72 and 0.86, respectively. Both of the latter two SNPs had been identified in BayeScan tests incorporating all four dog breeds.

**Fig 3:**
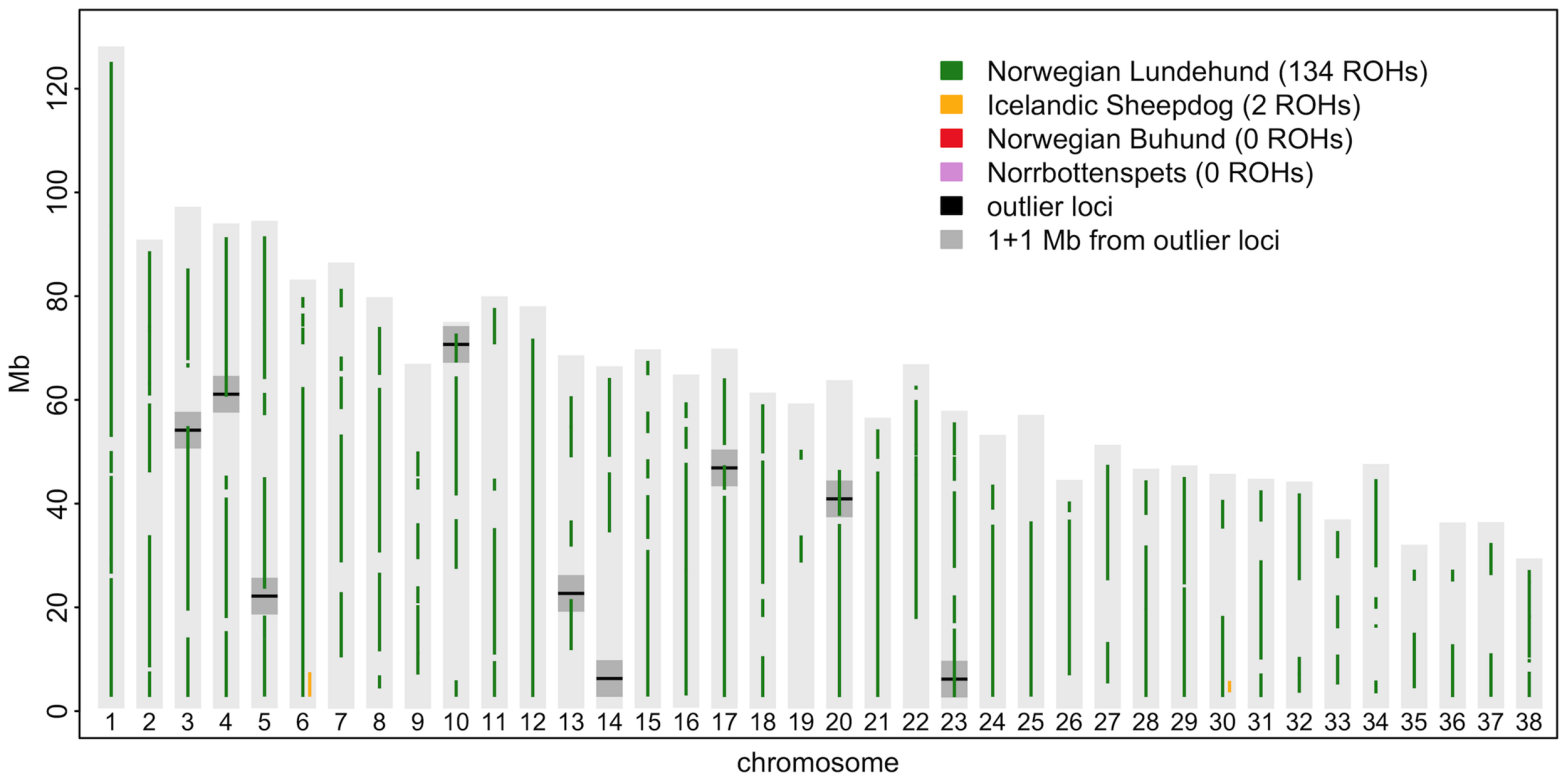
Runs of homozygosity (ROH) locations in the genome for four dog breeds. The genomic location of runs of homozygosity (ROH) shared by all individuals within each breed. Light grey bars outline the chromosomes, black lines denote the outlier loci from Bayescan analyses and dark grey marks flanking regions of the genome that were examined for genes under possible selection. Colored lines show the ROHs in each breed. On chromosome 30, Lundehund and Icelandic Sheepdog shared the same haplotype for their overlapping ROH, whereas on chromosome 6 they did not.

Genetic polymorphism values and mean ROH lengths per individual revealed a similar pattern (Table 1). Furthermore, ROH shared by all individuals covered approximately 2/3 of the Lundehund genome but were much rarer or absent in the other breeds (Fig 3). Concordantly, levels of IBD showed the opposite breed order, with very high values for the Lundehund, reduced levels in Buhund and Icelandic Sheepdogs, and lowest values for the Norrbottenspets. For ROH shared by all individuals in a breed we detected 134 in the Lundehund and two in the Icelandic Sheepdog, and we found none in the Buhund or Norrbottenspets (Fig 3). When considering ROH shared by 2/3 of the individuals within a breed there were still 134 in the Lundehund and none in the Norrbottenspets, though we observed 40 in the Icelandic Sheepdog and 32 in the Buhund (Figure C in S1 File). There was no obvious relationship between the distribution of ROH and outlier loci.

**Fig 4:**
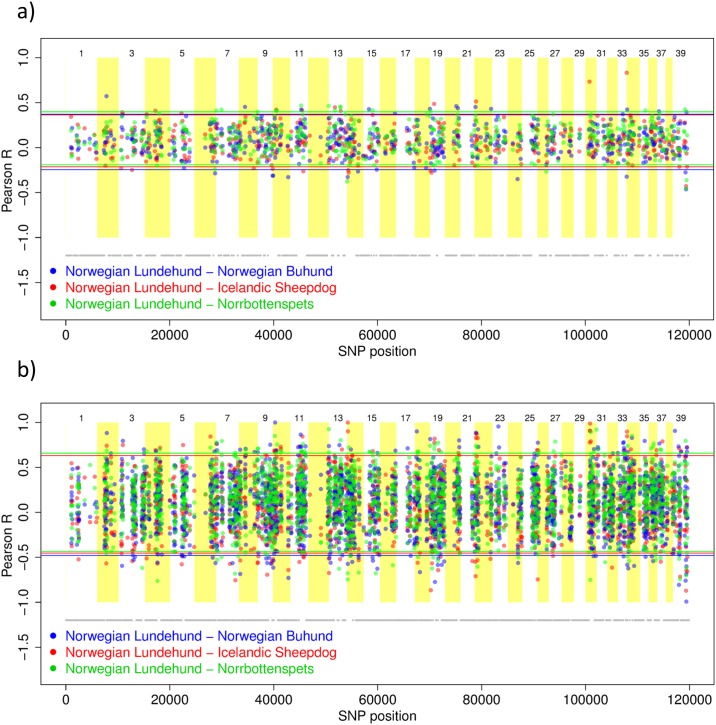
Pearson correlation coefficient between allele frequencies along the chromosome for four dog breeds. Norwegian Lundehund (n = 17), Norwegian Buhund (n = 10), Icelandic Sheepdog (n = 9) and Norrbottenspets (n = 12). Plots show the correlation coefficient (dots) with 95% confidence intervals (coloured lines) *versus* SNP position in sliding windows for a) every 100 SNPs and b) every 20 SNPs. Areas marked in grey below the plots show chromosomal regions of the Lundehund genome lacking genetic diversity. Gaps in the line thus reflect diversity although small gaps are not always visible.

**Fig 5:**
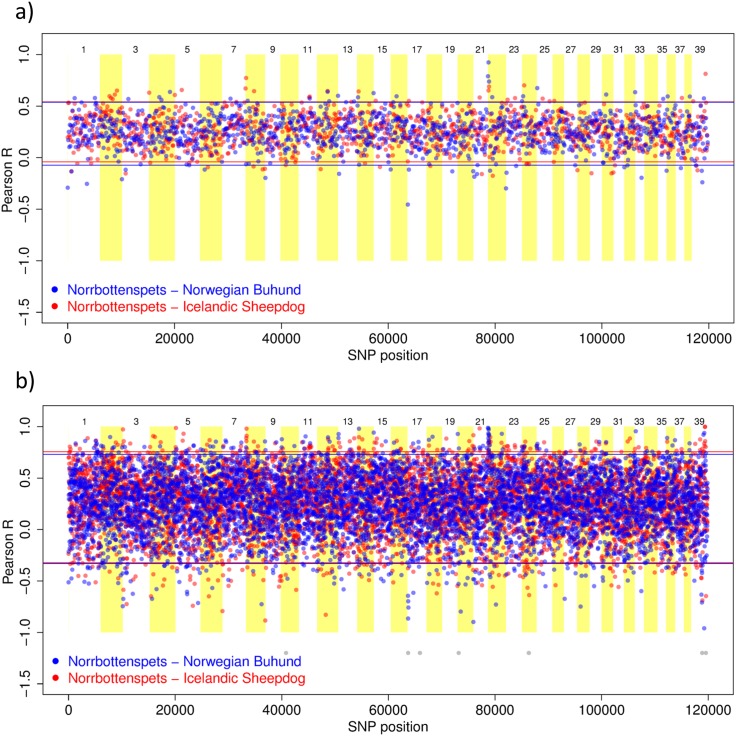
Pearson correlation coefficient between allele frequencies along the chromosome for three dog breeds. Norrbottenspets (n = 12), Norwegian Buhund (n = 10), and Icelandic Sheepdog (n = 9). We chose the Norrbottenspets as basis for comparison because this breed exhibited the highest genetic diversity. Plots show the correlation coefficient (dots) with 95% confidence intervals (coloured lines) *versus* SNP position in sliding windows for a) every 100 SNPs and b) every 20 SNPs.

Sliding window plots showed significant positive and negative allele frequency correlations between the Lundehund and each of the other breeds, and these were observed across the genome. SNPs positively correlated between the Lundehund and other breeds could suggest selection for similar features, whereas SNPs negatively correlated between breeds may indicate divergent selection or genetic drift within the breeds. Areas of apparent divergence were observed on chromosome 14 for the 100-SNP window analysis as well as on the X-chromosome (number 39) (Fig 4a, Figure D in S1 File). For the 20-SNP window analysis, other regions also emerged, including chromosome 18 (Fig 4b, Figure D in S1 File). Very limited genetic variability was observed on chromosomes 12 and 21. Significant negative and positive correlations were also found for analyses without the Lundehund (Fig 5a and 5b). In contrast, analyses of the Lundehund *versus* other breeds revealed extensive regions without allelic diversity. These areas are marked in grey colour below plots (Fig 4a and 4b; Figure D in S1 File) whereas breaks in the grey line show variable regions. Plots for expected and observed heterozygosity showed high overall values for the three candidate breeds, but markedly lower values for both parameters in the Lundehund (Figure E in S1 File). These results also indicate within-chromosome differences in the amount and distribution of genetic diversity among breeds.

## Discussion

Our results of low genetic diversity and high levels of inbreeding in the Lundehund are consistent with findings from earlier studies with a range of genetic markers [11–14]. Furthermore, the observed and expected heterozygosity are substantially lower in Lundehund than in the candidate breeds used for crossbreeding. The second-lowest variability was found in the Buhund. Based on our results, there are arguments supporting that introduction of Norrbottenspets genetic material in the Lundehund would result in more genetically variable offspring. The F_ST_ value between Lundehund and the Norrbottenspets was also lower than for other candidate breeds. In addition, ADMIXTURE results for K = 5–6 showed high within-breed variation in the Norrbottenspets, which seems consistent with the breed having an open stud book.

Although the Buhund and the Icelandic Sheepdog show relatively high within-breed relatedness and low genetic variation when compared with the Norrbottenspets, earlier studies suggest that the crossing of two groups with a high degree of inbreeding can provide substantial positive effects of genetic rescue, especially in the short term [32]. Importantly, several features can only be evaluated once the offspring of these crosses are older. For example, among the crossbred individuals, full siblings may have different morphology, behaviour and health conditions, which will influence selection of future breeding animals. Although the initial plans suggest to cross these individuals back into the Lundehund with use of purebred animals, further crossing among hybrids (e.g. Lundehund x Buhund with Lundehund x Icelandic Sheepdog) might be considered if health, morphology or other features indicate added benefits.

Despite the use of > 15K SNPs for F_ST_ estimates between Lundehund and other breeds, sample size differences could have affected the results as only few individuals were available for certain breeds. Additionally, the extent to which higher F_ST_ values imply that breeds have a genuinely *different* genetic composition caused by deliberate selection and/or genetic drift, or whether high values primarily reflect a lack of variation in the Lundehund, should be considered. Higher variation within breeds such as the Norrbottenspets could also have resulted in lower F_ST_ values in comparisons between this and other breeds, including the Lundehund. The F_ST_ estimates should therefore be interpreted with some caution. Notably, in preliminary ordination analyses by means of multidimensional scaling across 13 Nordic Spitz breeds (listed in Table A in S1 File), the Norrbottenspets appeared more differentiated from the Lundehund than the Buhund and Icelandic Sheepdog were (E. Salmela and H. Lohi, unpublished data); in addition, the genetic distance between the Lundehund and the Norrbottenspets was approximately equal to the distances between the Lundehund and several other Spitz breeds. The apparent difference between these and our PCA results (Fig 2), where Norrbottenspets appeared to be the closest of the candidate breeds to Lundehund, could relate to the different weight that the analyses give to the SNPs that differentiate between Lundehund and the three candidate breeds *versus* the SNPs that differentiate between Lundehund and the Nordic Spitzes in general. Despite the limitations inherent in the F_ST_ estimates, such comparisons are informative and also illustrate how genetics is but one component to be taken into account in a genetic rescue project that seeks to preserve a domestic breed with a unique morphology, behaviour and history.

Outlier SNPs were observed near genes associated with a variety of features important for health, development and morphology. The BayeScan analyses identified several outliers in pairwise comparisons of candidate breeds, and suggest there may be important diversity among the three candidate breeds, which was supported by the LOSITAN results. For example, *LRRTM1* on chromosome 17 has been linked to a rare phenotype associated with avoiding confined space in mice [29]. The gene was observed in the region flanking an outlier SNP between Buhund and Norrbottenspets, and has no known direct relevance for the Lundehund. However, the possibility that two candidate breeds may comprise important genetic variation relevant to characteristics such as behaviour in confined spaces may be valuable, as the Lundehund has been selected for affinity to closed spaces, i.e., for entering cavities with puffin nests. Crossbreeding with all three candidate breeds could therefore help alleviate inbreeding and optimize Lundehund genetic variation and evolutionary potential.

The comparisons across all four breeds indicated several outlier SNPs whereas no pairwise comparison with Lundehund was significant. The extreme loss of genetic diversity and high degree of genetic drift in the Lundehund, which appears to have had an effective population size of < 200 over the past 20 generations [12] could have contributed to this result. The relatively small sample size of each breed in our investigation may also have played a role, making the comparison involving all four breeds more powerful. There was no obvious relationship between ROH and outliers, which may be explained by outlier methods emphasising among-breed differences rather than extreme homozygosity within a single breed.

Sliding window plots of allele frequency correlations between the Lundehund and other breeds, and among the candidate breeds, showed negatively correlated SNPs that could suggest genetic drift or divergent selection. These results seem consistent with outlier analyses showing significant negative correlations between the Lundehund and other breeds, which appears to support cross-breeding with all candidate breeds to maximize genetic variation and long-term evolutionary potential. The analyses of heterozygosity are consistent with earlier results indicating a genome-wide paucity of genetic diversity for the Lundehund compared with other breeds.

### Conclusions and recommendations

Based on our results, we suggest crossbreeding with several candidate breeds to optimize long-term genetic diversity for the Lundehund and to reduce the incidence of serious health problems currently affecting the breed. This is in accordance with recommendations based on analyses of Lundehund pedigrees [33] that show extremely high relatedness of individuals within the breed. The results suggest that each candidate breed may contribute genetic diversity for different local regions within chromosomes, which could augment the long-term effect of the genetic rescue. This should also, over time, help reduce the extensive regions of the Lundehund genome lacking genetic variation. The outcrossing project provides a unique opportunity to combine genetic data with observed behaviour and morphology to guide the evolutionary trajectory and conservation of an endangered breed while balancing human-desired traits and natural selection.

As discussed above, it is challenging to balance the introduction of new genetic variation without swamping what remains of original genetic diversity. Notably, the decisions on which individuals to include in the cross-breeding program will not only be based on genetic information. Instead, careful observation and selection based on morphology, health status and behaviour is likely to play an important role in selecting individuals toward maintaining, as far as possible, the historical phenotype, even though the breed’s original and specialised duties as puffin hunters are no longer practically relevant. Introgression of new genetic, morphological and behavioural variation may broaden the phenotype, although this may be seen as an acceptable trade-off for preserving a severely bottlenecked and inbred population. These challenges are likely relevant across populations and species where genetic diversity and numbers of individuals have decreased to a point where managers are contemplating genetic rescue. Importantly, it may be valuable to select individuals while considering as baseline the historic phenotypic range of the breed, which appears to have been more diverse than that observed today including different coat colour patterns [10]. Additionally, future cross-breeding may be carefully managed to ensure that hybrids nearing the “limit” of the acceptable phenotype as determined by breed/species managers are crossed with a pure Lundehund, whereas hybrids closer to the standard could be bred with other similar hybrids to achieve a broader gene pool with a range of individuals that can be evaluated for morphology, behaviour and health condition.

Some might question the approach of selecting candidate breeds for outcrossing based on phenotypic information, and subsequent genetic analyses of the selected breeds only. However, as phenotypes (including morphology and behaviour) are often paramount for domestic breed owners and managers, such an approach could provide a helpful blueprint for genetic rescue and restoration of other domestic populations at risk by ensuring the active involvement of breeders and managers at the start of the project and taking advantage of their knowledge in the selection of candidate breeds for subsequent genetic investigation.

We strongly advocate that animals from this genetic rescue project are genotyped (animals from current and future generations) and that resources are devoted to following the project closely including phenotyping of animals. The Lundehund cross-breeding initiative has the potential to become an illustrative model of the potential of genetic rescue in domestic species. This project can serve as a model for how to use genomic information to guide breeding decisions in breeds that are genetically depauperate, and help illuminate the genetic architecture of complex diseases. Specifically, individuals resulting from the current outcrossing project may offer an opportunity for a future case-control study comparing disease-affected dogs with individuals showing good lifetime health records. This approach could be used for further investigation of *LEPREL1* (chromosome 34) and *NOD1* (chromosome 14) that were highlighted in a recent investigation of gastrointestinal disease in the Lundehund [34]. Such studies can similarly be undertaken to investigate polydactyly and its possible relationship with inbreeding and survival. Together, the Lundehund’s potential as a model organism for investigating the genetic basis for polydactyly [13] and the ongoing crossing project represent a chance to investigate the extent to which polydactyly may have negative effects in vertebrate development or whether health issues in breeds with this feature are independent consequences of inbreeding [17].

We recommend further research for the observed genes associated with characteristics that are part of the current breed standard (hypermobile joints, polydactyly) and may have implications for animal health. Although these genes are not, at present, considered to have direct implications for the Lundehund or the breeding program, future research could examine the possibility that the *GLB1* gene on chromosome 23 related to metabolism and hypermobile joints might represent a trade-off, whereby selection for hypermobile joints could have negative metabolic consequences by means of pleiotropic effects. The cross-breeding offspring from this project will likely show phenotypic variation for e.g. polydactyly and gastrointestinal health. These individuals can therefore contribute beyond the genetic rescue of an endangered breed toward case-control studies of phenotypic traits critical for vertebrate (including human) survival and health, thus improving our understanding of inbreeding, natural selection, and evolution.

## Supporting information

S1 File Supporting information with additional tables and figures. **Table A—**Pairwise F_ST_-values for the Lundehund and a selection of other Nordic Spitz breeds. **Table B—**SNPs with ID, chromosome and position (bp) from CanFam2 found as outliers.
**Figure A—**ADMIXTURE cross-validation error values for K (population clusters) from 1–6. **Figure B—**ADMIXTURE results for n = 41 Nordic dogs with 15 648 single nucleotide polymorphism loci and K = 2–6 population clusters.
**Figure C—**The genomic location of runs of homozygosity (ROH) shared by at least 2/3 of individuals within each breed.
**Figure D—**Pearson correlation coefficient between allele frequencies along the chromosome for the Lundehund and the candidate breeds.
**Figure E—**Expected and observed heterozygosity along the chromosome for the Lundehund and the candidate breeds.
(DOCX)
