# Supplementary material for: Genetic rescue of an endangered domestic animal through outcrossing with closely related breeds: A case study of the Norwegian Lundehund
Source: PLoS One. 2017 Jun 1;12(6):e0177429. doi: 10.1371/journal.pone.0177429 (PMC5453418; doi:10.1371/journal.pone.0177429)

**Supporting Information for ”Genetic Rescue of an Endangered Domestic Animal Through Outcrossing with Closely Related Breeds: a Case Study of the Norwegian Lundehund”**

Table A. Pairwise F_ST_-values for the Lundehund (n=17) and a selection of other Nordic Spitz breeds based on individual genotype data for 15K single nucleotide polymorphism (SNP) loci. Values are presented as unweighted and weighted according to Weir and Cockerham (1984)^1^ with p-value in parentheses and the number of permutations and breed sample size.

| **Breed** | **F_ST_-value unweighted (p-value)** | **F_ST_-value weighted (p-value)** | **Permutations** | **Sample size** |
| --- | --- | --- | --- | --- |
| Norwegian Elkhound Grey | 0.280 (0.0025) | 0.456 (0.0025) | 396 | 14 |
| Swedish Elkhound | 0.297 (0.0027) | 0.446 (0.0027) | 376 | 9 |
| Swedish White Elkhound | 0.465 (0.0027) | 0.616 (0.0027) | 373 | 4 |
| Swedish Vallhund | 0.357 (0.0012) | 0.542 (0.0012) | 812 | 9 |
| Finnish Spitz | 0.205 (0.0052) | 0.351 (0.0026) | 385 | 36 |
| Finnish Lapphund | 0.128 (0.0225) | 0.222 (0.0056) | 354 | 62 |
| Karelian Beardog | 0.211 (0.0025) | 0.358 (0.0025) | 403 | 25 |
| Laika | 0.322 (0.0026) | 0.448 (0.0026) | 382 | 6 |
| West Siberian Laika | 0.329 (0.0028) | 0.455 (0.0028) | 360 | 6 |
| East Siberian Laika | 0.325 (0.0026) | 0.450 (0.0026) | 386 | 6 |
| Norwegian Buhund | 0.228 (0.0027) | 0.381 (0.0027) | 363 | 10 |
| Icelandic Sheepdog | 0.247 (0.0055) | 0.410 (0.0082) | 364 | 9 |
| Norrbottenspets | 0.166 (0.0054) | 0.263 (0.0054) | 367 | 12 |

^1^Weir BS, Cockerham CC. Estimating F-statistics for the analyses of population structure. Evolution. 1984; 38:1358–1370.

Table B. SNPs with ID, chromosome and position (bp) from CanFam2 found as outliers in BayeScan analyses across Lundehund (n=17), Norwegian Buhund (n=10), Icelandic Sheepdog (n=9) and Norrbottenspets (n=12).

| **SNP ID** | **Chromosome and position (bp)** | **Comparison^1^** |
| --- | --- | --- |
| BICF2S23152168 | chr3:54160905 | 4B |
| BICF2P480043 | chr4:61093673 | 4B, BN |
| BICF2P261357 | chr5:22167407 | 4B |
| BICF2P663203 | chr10:70696871 | 4B |
| BICF2P1052982 | chr13:22691061 | BN |
| BICF2G630518309 | chr14:6266321 | IN |
| BICF2G630518318 | chr14:6283930 | IN |
| BICF2P993491 | chr17:38404232 | 4B |
| TIGRP2P232559_rs8827295 | chr17:46888130 | BN |
| BICF2P941107 | chr20:40918366 | 4B |
| BICF2S2334188 | chr21:22386123 | BN |
| BICF2P719133 | chr22:31951671 | 4B |
| TIGRP2P309625_rs8576070 | chr23:6159976 | 4B, BN, IN |
| BICF2G630155141 | chr27:47425503 | BN |
| BICF2P598467 | chr27:47444976 | BN |
| BICF2P17760 | chr27:48062576 | 4B, BI, BN |
| BICF2P1275372 | chr27:48093163 | BI |
| BICF2S23226849 | chr34:42287893 | 4B |

^1^Pairwise breed comparisons for: B – Buhund; N – Norrbottenspets; I – Icelandic Sheepdog. 4B: across all four dog breeds, including the Lundehund.

Figure A. ADMIXTURE cross-validation error values for K (population clusters) from 1-6, showing support (i.e., lowest error value) for K = 2 (Lundehund *versus* the three other breeds Buhund, Icelandic Sheepdog and Norrbottenspets) followed by K = 4 (all breeds as separate clusters), as shown in Fig 1. Increasing K-values beyond 4 showed within-breed variation in the Norrbottenspets.


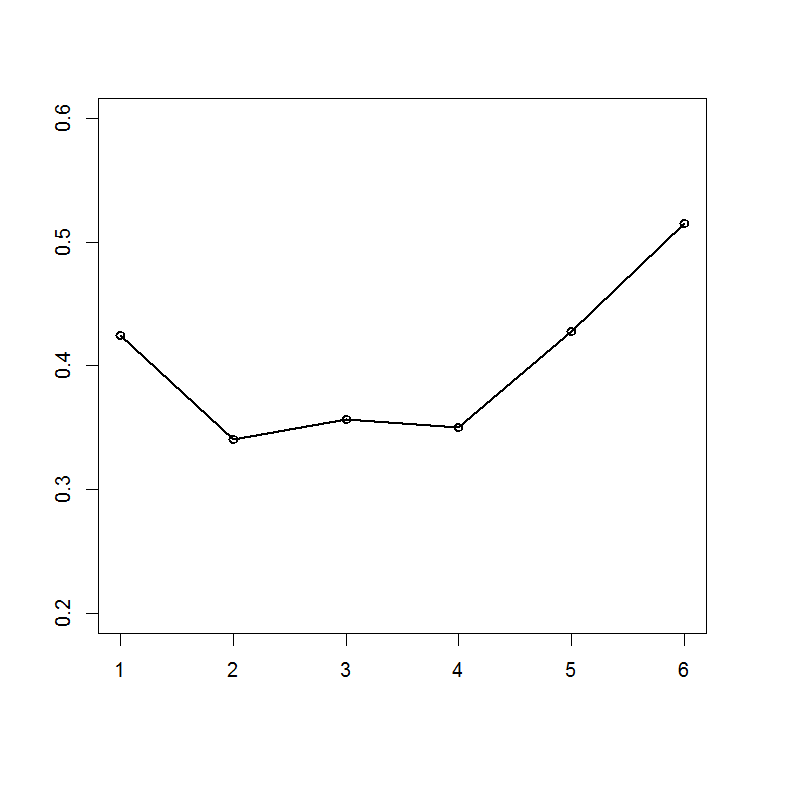


Cross-validation error

K-value (number of proposed population clusters)

Figure B. ADMIXTURE results for n = 41 Nordic dogs with 15 648 single nucleotide polymorphism (SNP) loci and K = 2 (top) to K = 6 (bottom) population clusters for the four breeds Norwegian Lundehund (LUN), Norwegian Buhund (BUH), Icelandic Sheepdog (ICE) and Norrbottenspets (NOR), where 10 Lundehund-individuals were selected randomly to examine clustering with equalized sample sizes. Comparison of cross-validation errors showed highest support for K = 2 followed by K = 4.


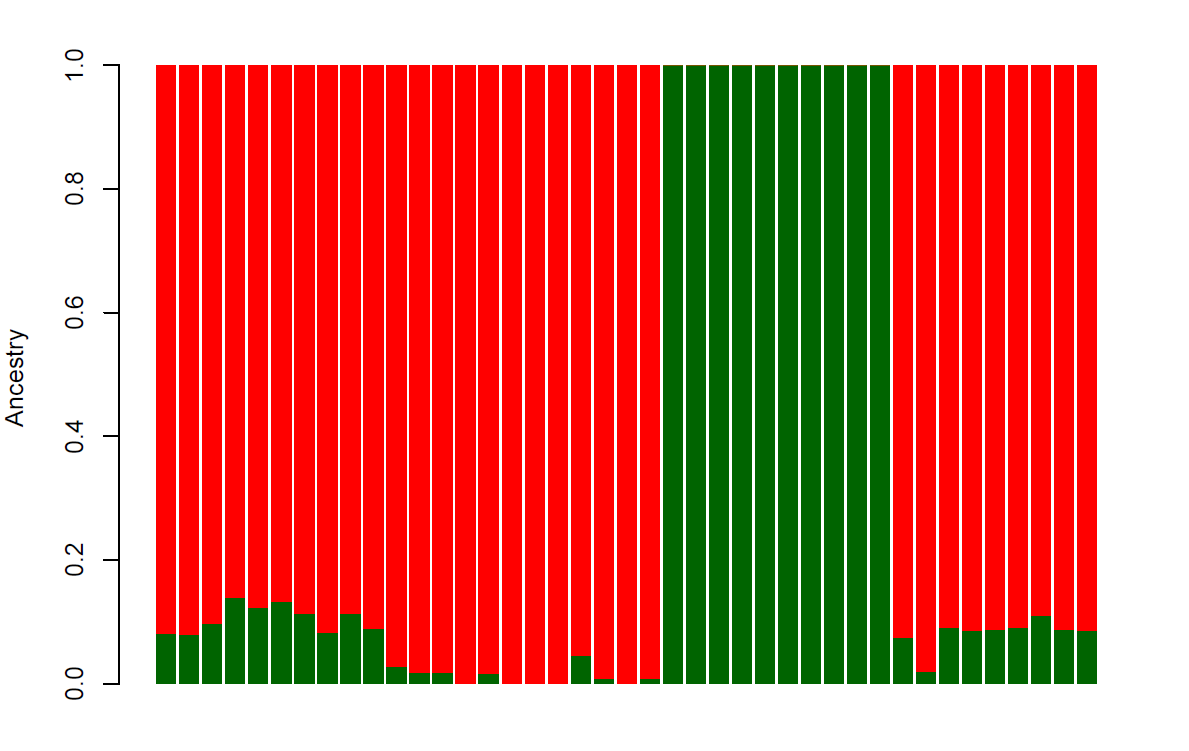


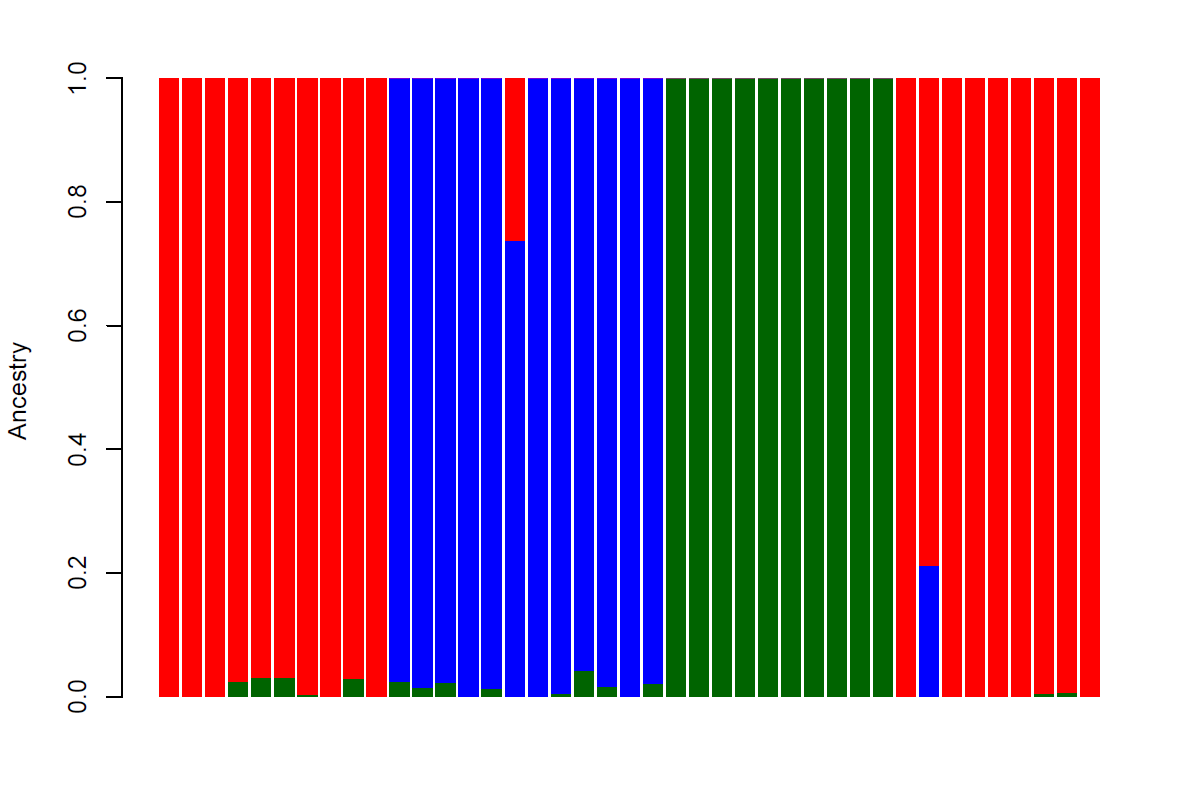


|------- BUH -------| ----------- NOR -------- | ------- LUN -------- | ------ ICE ---------|


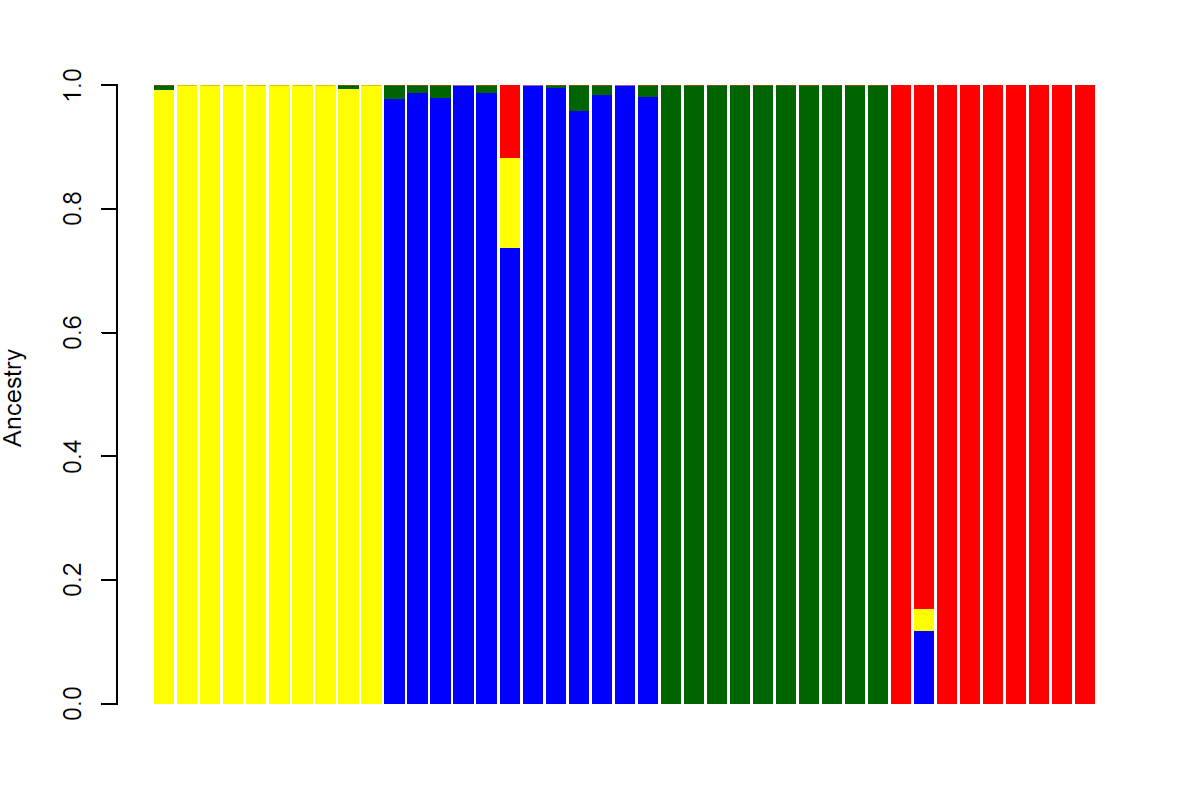


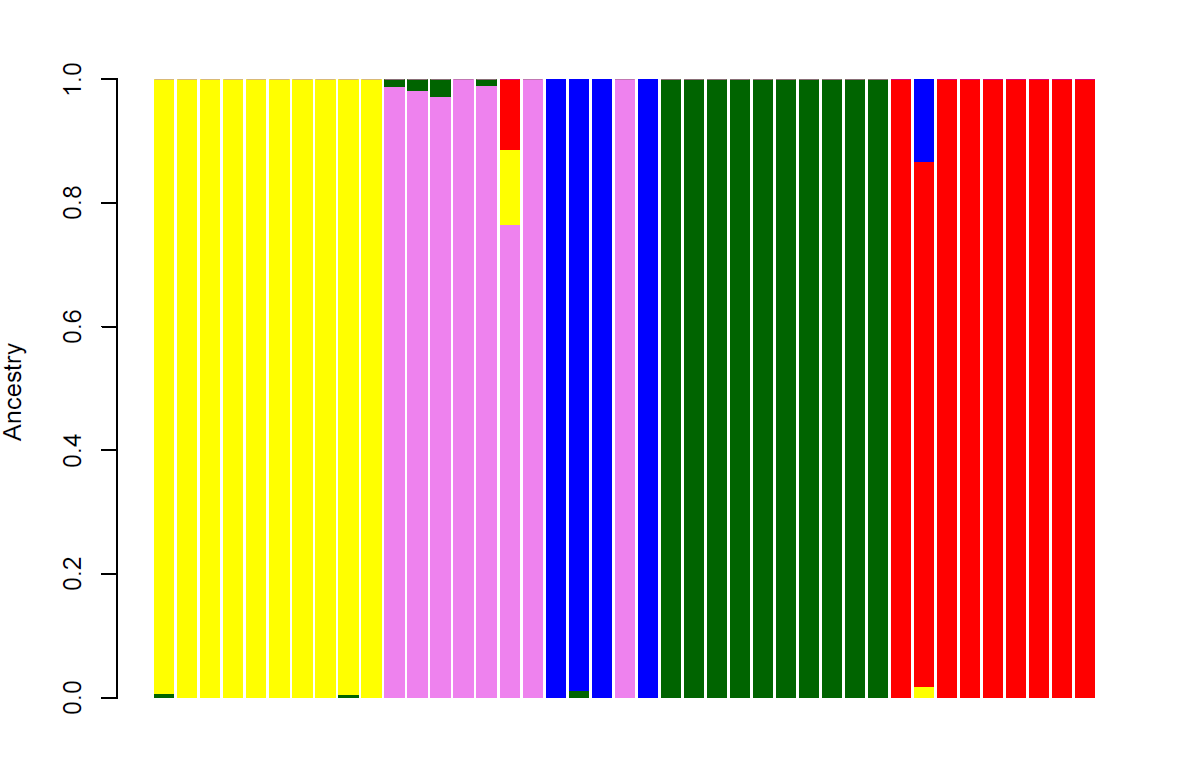


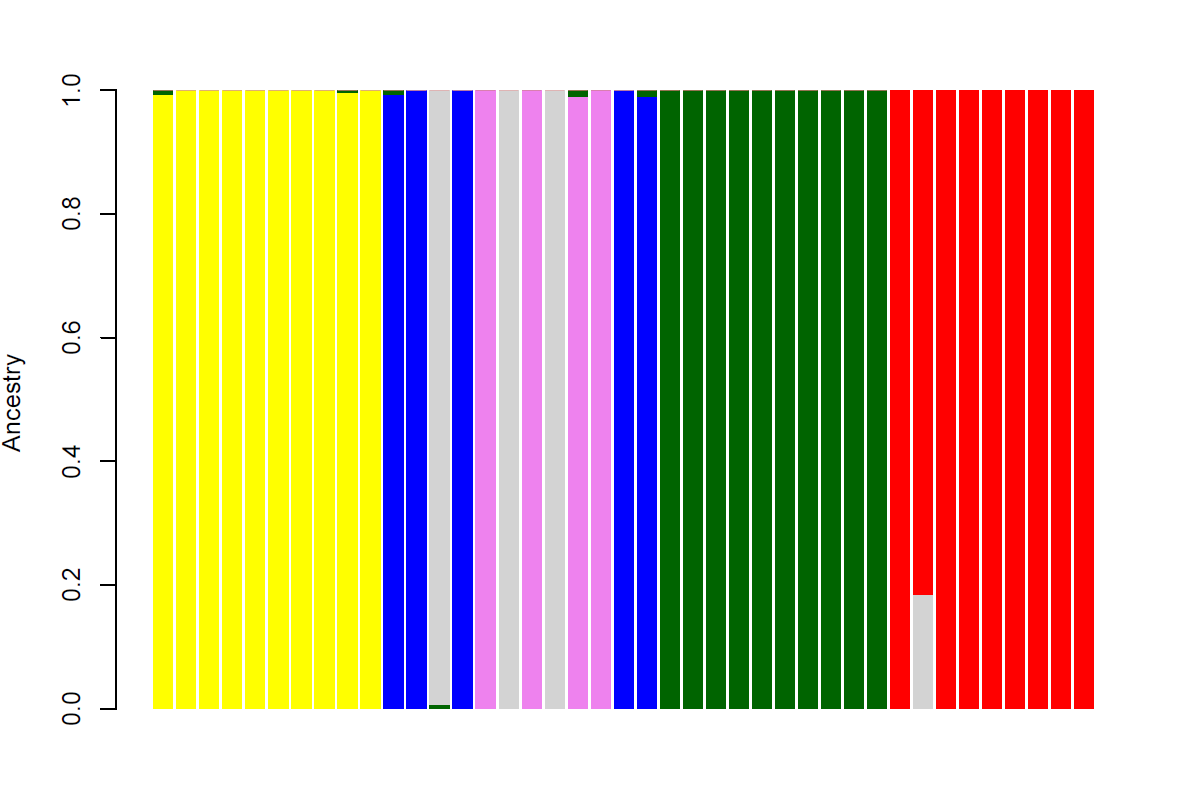


|------- BUH ------- | ----------- NOR --------- | -------- LUN -------- | ------ ICE ---------|

Figure C. The genomic location of runs of homozygosity (ROH) shared by at least 2/3 of individuals within each breed. Light grey bars outline the chromosomes, black lines denote the outlier loci from Bayescan analyses and dark grey marks flanking regions of the genome that were examined for genes under possible selection. Colored lines show the ROHs in each breed.


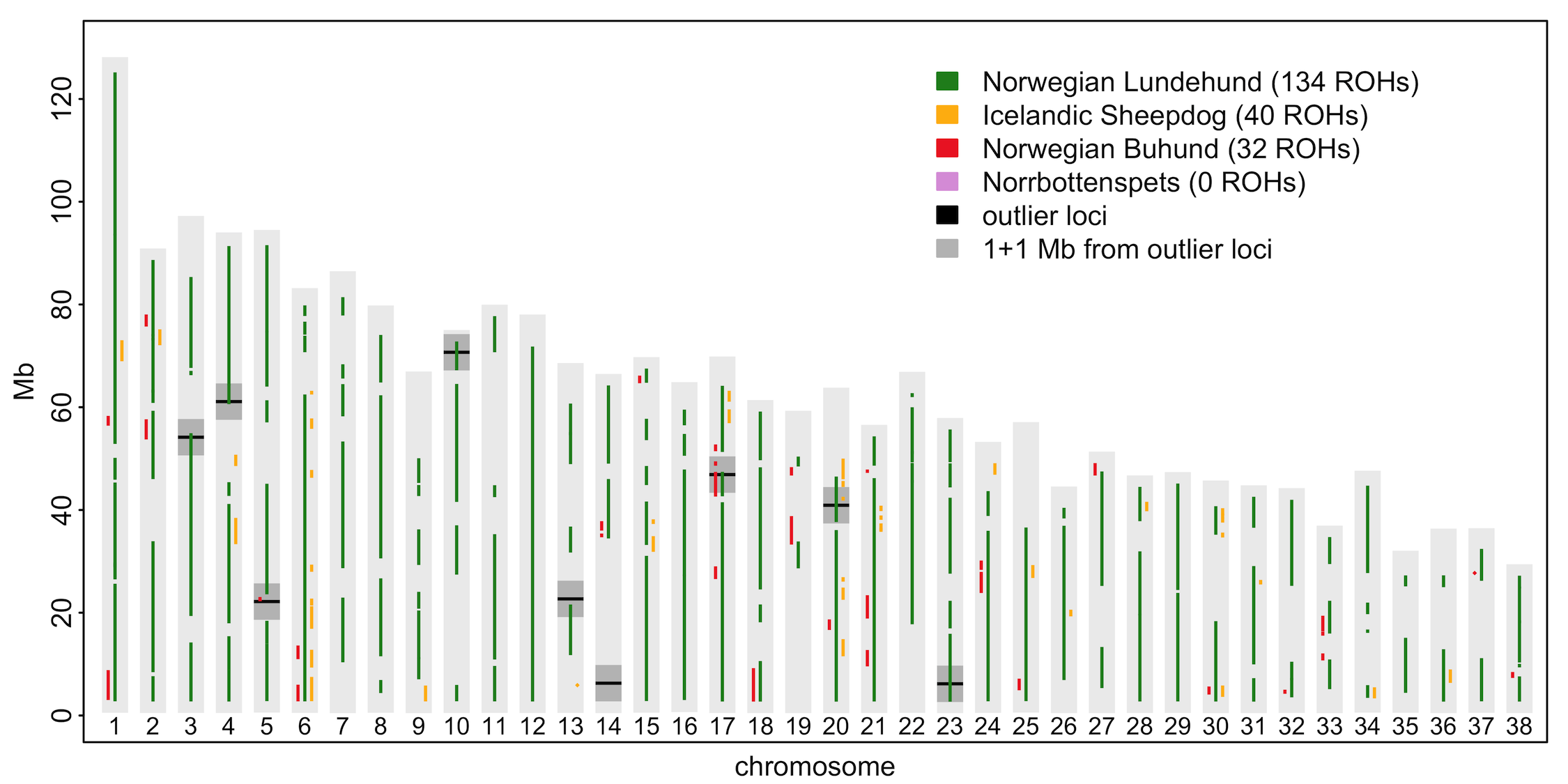


Figure D. Pearson correlation coefficient between allele frequencies along the chromosome for the Norwegian Lundehund (n = 17), the Norwegian Buhund (n = 10), the Icelandic Sheepdog (n = 9) and the Norrbottenspets (n = 12). Plots illustrate segments of Fig 4, showing the Pearson correlation coefficient (dots) with 95% confidence intervals (coloured lines) versus SNP position in sliding windows for every 20 SNPs within each chromosome, showing details of a) chromosomes 10-15 and b) chromosomes 17-21 (every second chromosome is shown in yellow). Areas marked in grey below the plots show chromosomal regions of the Lundehund genome lacking genetic diversity. Gaps in the line thus reflect diversity (though small gaps are not always visible).


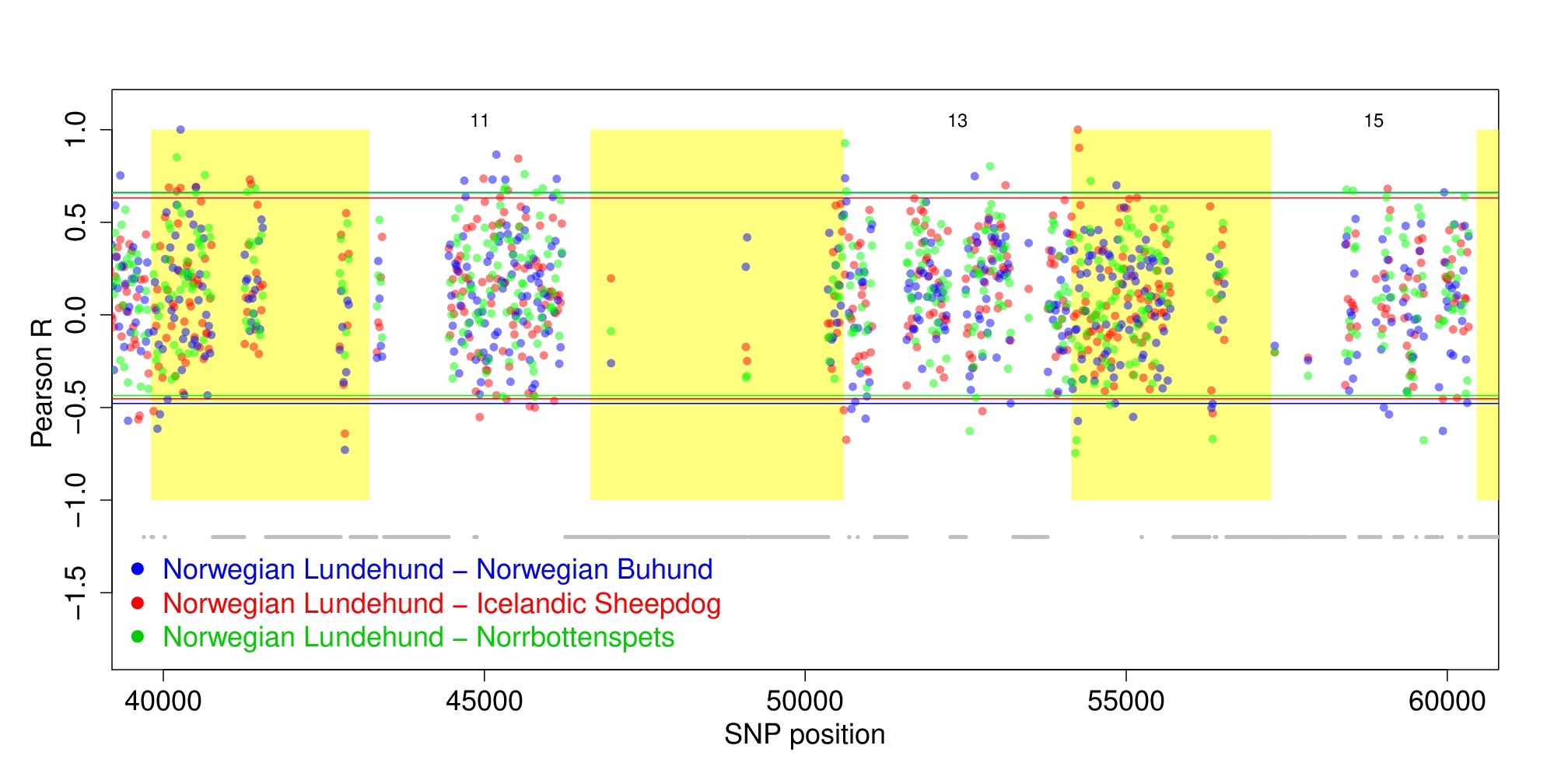


**
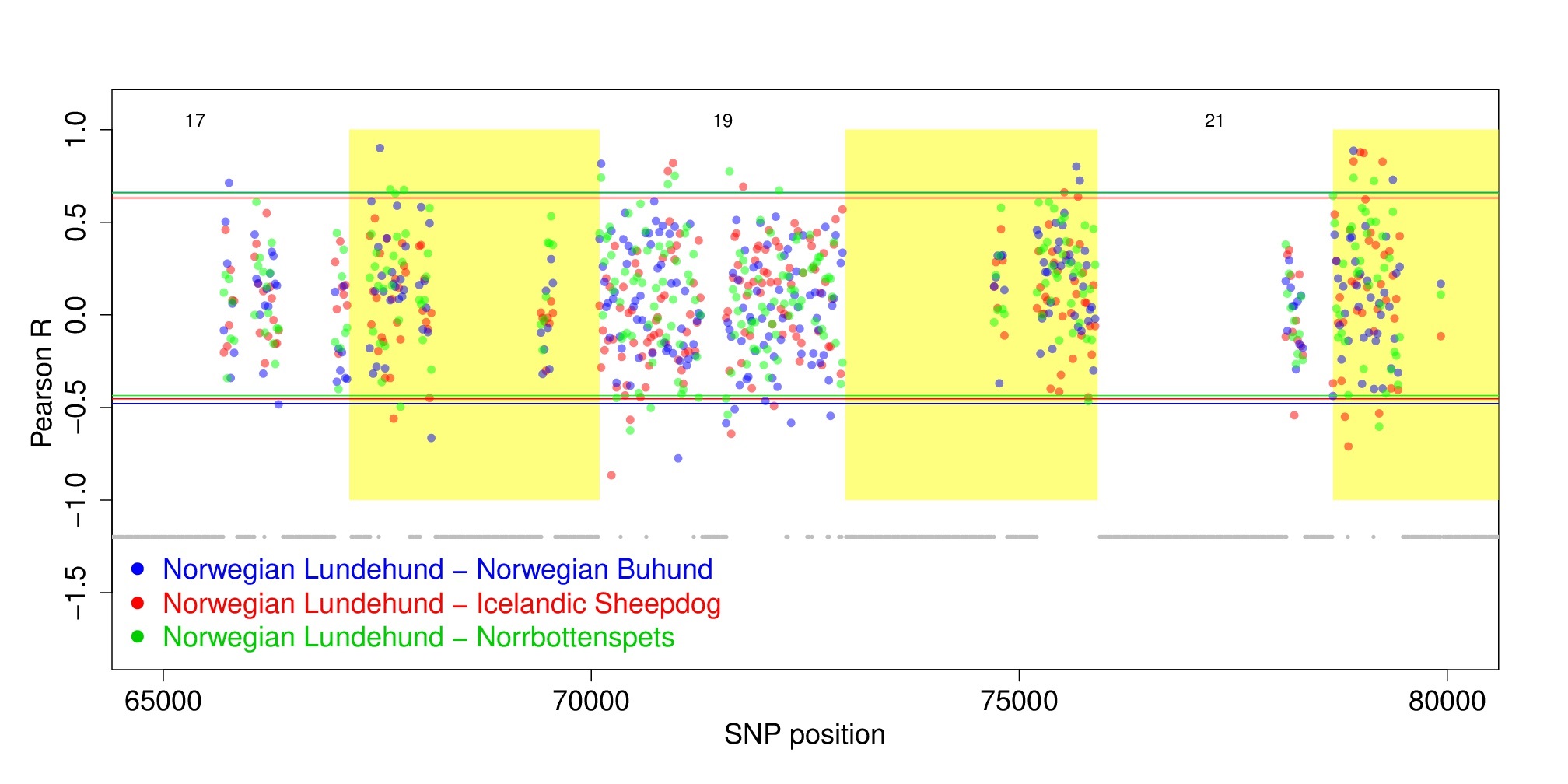
**

Figure E. Expected and observed heterozygosity along the chromosomes for the Norwegian Lundehund (n = 17), the Norwegian Buhund (n = 10), the Icelandic Sheepdog (n = 9) and the Norrbottenspets (n = 12).


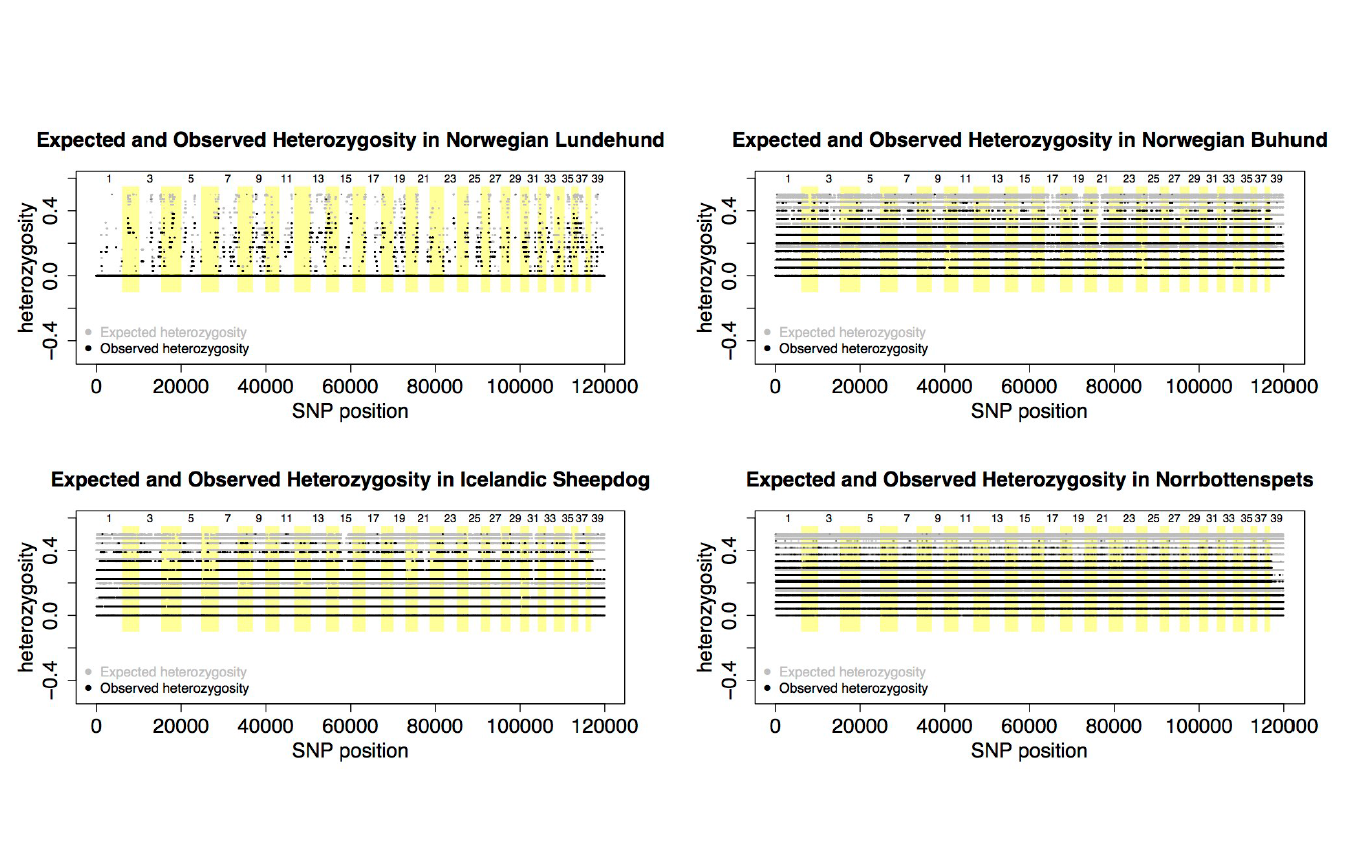

Supplement: S1 File — Table A—Pairwise FST-values for the Lundehund and a selection of other Nordic Spitz breeds. Table B—SNPs with ID, chromosome and position (bp) from CanFam2 found as outliers. Figure A—ADMIXTURE cross-validation error values for K (population clusters) from 1–6. Figure B—ADMIXTURE results for n = 41 Nordic dogs with 15 648 single nucleotide polymorphism loci and K = 2–6 population clusters. Figure C—The genomic location of runs of homozygosity (ROH) shared by at least 2/3 of individuals within each breed. Figure D—Pearson correlation coefficient between allele frequencies along the chromosome for the Lundehund and the candidate breeds. Figure E—Expected and observed heterozygosity along the chromosome for the Lundehund and the candidate breeds. (DOCX) [file pone.0177429.s001.docx]
